# Systematic review and meta-analysis for the occurrence of Crimean-Congo hemorrhagic fever, Tularemia, and Rift Valley fever in pastoralist systems in Africa

**DOI:** 10.3389/fvets.2025.1624748

**Published:** 2026-03-31

**Authors:** Dickson Machira Nyaguthii, James Hassel, Daniel Nthiwa, Michael E. von Fricken, Ellen P. Carlin, Francesco Fava, Victor Ofula, Dino Martins, David Redding, Jeffrey W. Koehler, Mathew Muturi, Eric M. Fèvre, Peter Kimeli, Joshua Orungo Onono

**Affiliations:** 1 Health Theme, International Livestock Research Institute, Nairobi, Kenya; 2 Faculty of Veterinary Medicine, University of Nairobi, Nairobi, Kenya; 3 Smithsonian’s Global Health Program, Washington, DC, United States; 4 Department of Biological Sciences, University of Embu, Embu, Kenya; 5 One Health Center of Excellence, College of Public Health and Health Professions, University of Florida, Gainesville, FL, United States; 6 Parapet Science and Policy Consulting, Washington, DC, United States; 7 Department of Environmental Science and Policy, Università degli Studi di Milano, Milan, Italy; 8 Kenya Medical Research Institute, Nairobi, Kenya; 9 Turkana Basin Institute, Stony Brook University, Stony Brook, NY, United States; 10 Natural History Museum, London, United Kingdom; 11 Diagnostic Systems Division, United States Army Medical Research Institute of Infectious Diseases, Fort Detrick, MD, United States; 12 Zoonotic Disease Unit, State Directorate of Veterinary Services, Nairobi, Kenya; 13 Department of Veterinary Medicine, Dahlem Research School of Biomedical Sciences (DRS), Freie Universität Berlin, Berlin, Germany; 14 Institute of Infection, Veterinary, and Ecological Sciences, University of Liverpool, Liverpool, United Kingdom

**Keywords:** zoonotic, systematic literature review, pastoralism, Rift Valley fever, Crimean-Congo hemorrhagic fever

## Abstract

**Introduction:**

Pastoralism is a livestock production system practiced in areas with harsh environmental conditions and is characterized by low investment in animal health, increasing the risk of animal disease outbreaks. Zoonotic diseases such as Crimean-Congo hemorrhagic fever (CCHF), Rift Valley fever (RVF), and tularemia may spread through livestock product value chains.

**Methods:**

Published studies on the three diseases were systematically searched in PubMed, Web of Science, Scopus, and Google Scholar using predetermined search terms. Eligible studies were screened, and full texts were reviewed for data extraction. Extracted variables included publication details, laboratory methods, and measures of disease occurrence. Studies were grouped according to livestock value chain nodes. Random-effects models were used to estimate pooled prevalence. Publication bias was assessed using Egger’s test and funnel plot symmetry. A random forest algorithm identified relevant moderators of prevalence, which were further evaluated using mixed-effects models.

**Results:**

A total of 34 studies were included, with RVF being the most studied pathogen (64.7%), followed by CCHF (29.4%) and tularemia (5.9%). RVF prevalence was highest in humans (29%; 95% CI: 7–69%), followed by camels (19%; 95% CI: 7–43%), and lowest in goats (6%; 95% CI: 4–10%). CCHF prevalence was highest in camels (48%; 95% CI: 8–91%) and lowest in humans (6%; 95% CI: 2–19%). At the value chain level, prevalence was highest at livestock farms for both RVF (13%; 95% CI: 10–16%) and CCHF (15%; 95% CI: 4–44%). Females were more likely to test positive (OR = 5.20; 95% CI: 3.09–8.76; *p* < 0.01), while mixed herds showed higher likelihood of RVF positivity (OR = 33.34; 95% CI: 0.72–1548.64; *p* = 0.0734). Tularemia had a pooled positivity rate of 2% (95% CI: 0–8%).

**Discussion:**

This review provides evidence that CCHF, RVF, and tularemia are endemic in pastoral areas. Strengthened surveillance along livestock value chain nodes is needed to mitigate the risk of disease transmission.

## 1 Introduction

Pastoralism is widely practiced in Africa as a form of adaptation to arid environmental conditions by livestock-keeping communities (1). The outputs from animals kept under pastoralist production systems contribute significantly to the African national economies and household food security (2). Livestock keeping is often low-input, extensive, and characterized by various forms of mobility (short- or long-range). This leads to frequent contact between domestic animals and wildlife. Often, herds are of mixed species, including cattle, sheep, goats, camels, and sometimes poultry. Due to frequent transboundary movements, limited investment, and budgetary constraints in host countries, animal health management among these populations remains inadequate. The combination of these factors may increase the risk of outbreaks of animal diseases, including zoonoses, which could impact the consumers of livestock products from these systems (3).

Value chains refer to pathways that a product follows from production to the final consumers, the actors involved, and the processes the product undergoes, which may not change its value before reaching the final consumer (4). Animal product value chains from this system are often short, with milk, meat, and eggs consumed locally, while live animals may be sold to markets in urban areas within or outside the host country (5, 6). An understanding of the role of actors and risks of zoonotic disease risks in each value chain node of the pastoralist livestock product value chains may inform the formulation of community-friendly and evidence-based control measures at specific points. Africa has 54 countries and an estimated population of 1.5 billion (7). However, most African countries have limited preparedness for zoonotic disease management (8).

Crimean-Congo hemorrhagic fever virus (CCHFV), Rift Valley fever virus (RVFV), and *Francisella tularensis* (Ft) are vector-borne zoonotic pathogens that occur in pastoral systems and can thus serve as representative zoonotic diseases spread along value chains (9). RVF, caused by the RVFV (*Phlebovirus riftense*) in the family *Bunyaviridae,* is endemic in Africa, being reported in over 80% of the African countries (10). The disease is spread by mosquitoes or by direct contact with contaminated animal tissues or secretions. CCHF is caused by the CCHFV (*Orthonairovirus haemorrhagiae*) in the family *Nairoviridae* and is mostly transmitted by ticks of the *Hyalomma* genus or through contact with tissues from infected animals (11). Around two-thirds of the countries in Africa do not have robust surveillance systems for CCHF, but the disease is thought to be present (12). Tularemia is a zoonotic disease caused by the highly infectious gram-negative coccobacilli Ft (13). In Africa, as elsewhere in the world, mosquitoes, biting flies, and ticks may serve as vectors (14). Transmission to humans may be by inhalation, ingestion of contaminated food or water, vectors, or through direct contact with infected animals or their tissues (15).

Livestock diseases can have major impacts on pastoralist livelihoods due to associated morbidity and mortality. This leads to additional direct costs for treatment and prevention of diseases, loss of animal products, and indirect costs such as those associated with changes to livestock movement patterns due to disease and loss of markets (16). Zoonotic livestock diseases also pose a risk to human health through direct contact with infected animals, vectors, or animal products. Epidemics of RVF have been reported in East Africa (10), West Africa, Southern Africa (17), and Northern Africa (18) and have been associated with human and animal mortalities in addition to disruptions of livestock economies. Outbreaks of CCHF have been reported in over a third of countries in Africa and have been associated with human mortality (12). Outbreaks of tularemia have been associated with significant morbidity and rarely mortality in animals and human beings (19–21). While RVF has been shown to cause death in humans and animals and disrupt value chains in pastoralist systems (22), the socioeconomic effects of CCHF and tularemia have not been investigated for pastoralist systems. The three diseases occurring at the production nodes (farms) can usually precede outbreaks in human beings who may be involved in animal handling or milk and meat processing (middlemen node) through direct contact with contaminated products or vectors. Furthermore, contaminated products reaching the consumer node may lead to outbreaks. Thus, an understanding of the baseline occurrence of these diseases is needed from both policy and disease control perspectives (23).

The objective of this meta-analysis, therefore, was to determine the baseline occurrence of CCHF, RVF, and tularemia in the pastoral landscapes of Africa and evaluate the risk of spread along livestock value chains. The central question the systematic review and meta-analysis was designed to answer was: do livestock product value chains influence the risk of spread of CCHF, RVF, and tularemia in pastoral systems in Africa?

## 2 Methodology

### 2.1 Eligibility and search criteria

The inclusion criteria were non-review papers that described livestock products originating in pastoral livestock production systems in Africa, where livestock products could be linked to a value chain node, where investigators used at least one laboratory diagnostic method in their methodology, and that described CCHF, RVF, and tularemia; and papers published since 2004. Papers describing livestock products from other livestock production systems (except pastoralism), studies not conducted in Africa, zoonotic diseases, or published before 2004 were excluded.

The databases searched were PubMed, Web of Science, Scopus, and Google Scholar. The search terms are shown in Table 1.

**Table 1:** Search terms and databases searched.

Database	Search terms	Number of publications in the result
PubMed	(pastoral* OR “extensive production system” OR Nomadic*) AND (Africa) AND (“Crimean Congo hemorrhagic fever” OR “Rift Valley Fever” OR RVF OR CCHF OR Tularemia OR “*Francisella tularensis*”)	49
Web of science	TS = (pastoral* OR “extensive production system” OR Nomadic*) AND TS = (Africa) AND TS = (“Crimean Congo hemorrhagic fever” OR “Rift Valley Fever” OR RVF OR CCHF OR Tularemia OR “*Francisella tularensis*” OR zoonos*) NOT TS = (review)	52
Scopus	(pastoral* OR “extensive production system” OR Nomadic*) AND (Africa) AND (“Crimean Congo hemorrhagic fever” OR “Rift Valley Fever” OR RVF OR CCHF OR Tularemia OR “*Francisella tularensis*” OR Zoonos*)	24
Google Scholar	Africa AND pastoral * “Crimean Congo haemorrhagic fever” OR CCHF OR “Rift Valley Fever” OR RVF OR Tularemia OR “*Francisella tularensis*” -review	484

### 2.2 Study selection

Abstracts and titles of studies were downloaded from the search databases and imported into Rayyan^1^ for removal of duplicates and examination of their eligibility for inclusion. The abstracts of the studies were examined by DMN and DN independently, and a decision was made on whether to include or exclude the studies based on eligibility criteria. Disagreements were resolved by consensus. For articles not in English, the titles and abstracts were translated using Google Translate and examined for eligibility. The full texts of the included articles were downloaded and examined.

### 2.3 Data extraction

Data were extracted from the papers using a data extraction tool. The data extraction tool contained the following variables: paper ID, year of publication, type of study, whether the paper investigated CCHFV, RVFV, or *Ft*, the animal product investigated, the country of study, the year of study, the sample size, a bias risk assessment, whether there was any effect size measured, the value of effect size including estimates of uncertainty around the effect size, the laboratory method used, and the animal species involved. The laboratory methods described in the papers were classified as direct pathogen detection (PCR, antigen detection methods, etc.) and prior exposure methods (IgG/IgM).

For studies not explicitly investigating value chains, any study reporting the occurrence of a pathogen in livestock was classified as occurring in the production node. Any study reporting the occurrence of a pathogen in human beings not explicitly stating their involvement in animal keeping or animal product processing but associated with pastoral livestock production systems was described as belonging to the consumer node, with all others classified according to their stage of animal keeping or animal product processing.

### 2.4 Quality assessment

To assess the quality of findings of the papers included, a bias risk assessment was conducted through a modified GRADE assessment tool. The components of the tool included randomization in the selection of participants, sample size determination, laboratory testing of samples, and whether the samples underwent peer review. One point was allocated to each component, and an overall score was calculated in a variable called bias risk.

### 2.5 Data analysis

To determine if the effect size (prevalence, incidence, or positivity) reported differed by pathogen, species, and value chain node, a meta-analysis was conducted using R’s meta package, with the *metaprop* function being used to summarize the effect sizes. The prevalence proportions reported in the studies were logit-transformed to calculate the overall proportions. Prediction intervals were calculated based on the t distribution. Furthermore, the *metagen* function was used to summarize odds ratios for reported risk factors across different studies. For both functions, a random effects model was utilized because of possible heterogeneity in the studies, with the inverse variance method used to pool the studies. A restricted maximum-likelihood estimator was used for tau^2,^ while a Q-Profile method for estimating the confidence interval of tau^2^ and tau was used. For studies that did not report 95% confidence intervals alongside their reported effect sizes, this was calculated using the standard approximation to the binomial distribution using their effect size and sample size.

An Egger’s regression test and symmetry of funnel plots were used to investigate publication bias (24). When more than three studies are available and significant bias was present, the *trimfill* function was used to estimate the effects of missing studies. For estimates with fewer than three studies, no correction was applied.

A machine learning model, random forests (25), was used to identify relevant predictors (moderators) of the effect size (prevalence) for each pathogen using the *Metaforest* function (26). The moderators tested for study year, species reported by the study, country of publication, bias risk estimate, laboratory method used in the publication, and value chain node for each pathogen. The most relevant moderators were selected based on variable importance. The moderator with the least importance was dropped, and reanalysis was done until only moderators with positive importance were left. This analysis was replicated 100 times using the *preselect* function. The moderators with positive variable importance in more than 45% of replications were then selected. The selected moderators were then put as predictors into a mixed effects model with the prevalences reported for each pathogen as the outcome.

Data was visualized using forest plots and tables. All other outcomes were summarized as narratives, proportions, tables, and graphs. Maps were drawn using QGIS Geographic Information System version 3.32.3 (Open-Source Geospatial Foundation Project). The Africa shape file used was created by the Intergovernmental Authority on Development (IGAD) in the Eastern Africa Climate Prediction and Applications Center (ICPAC). It contained administrative-level data on Africa. The attribute table was updated with publication counts by country, which were then visualized using graduated colors to show increasing values.

## 3 Results

### 3.1 Search results and study selection

**Figure 1:**
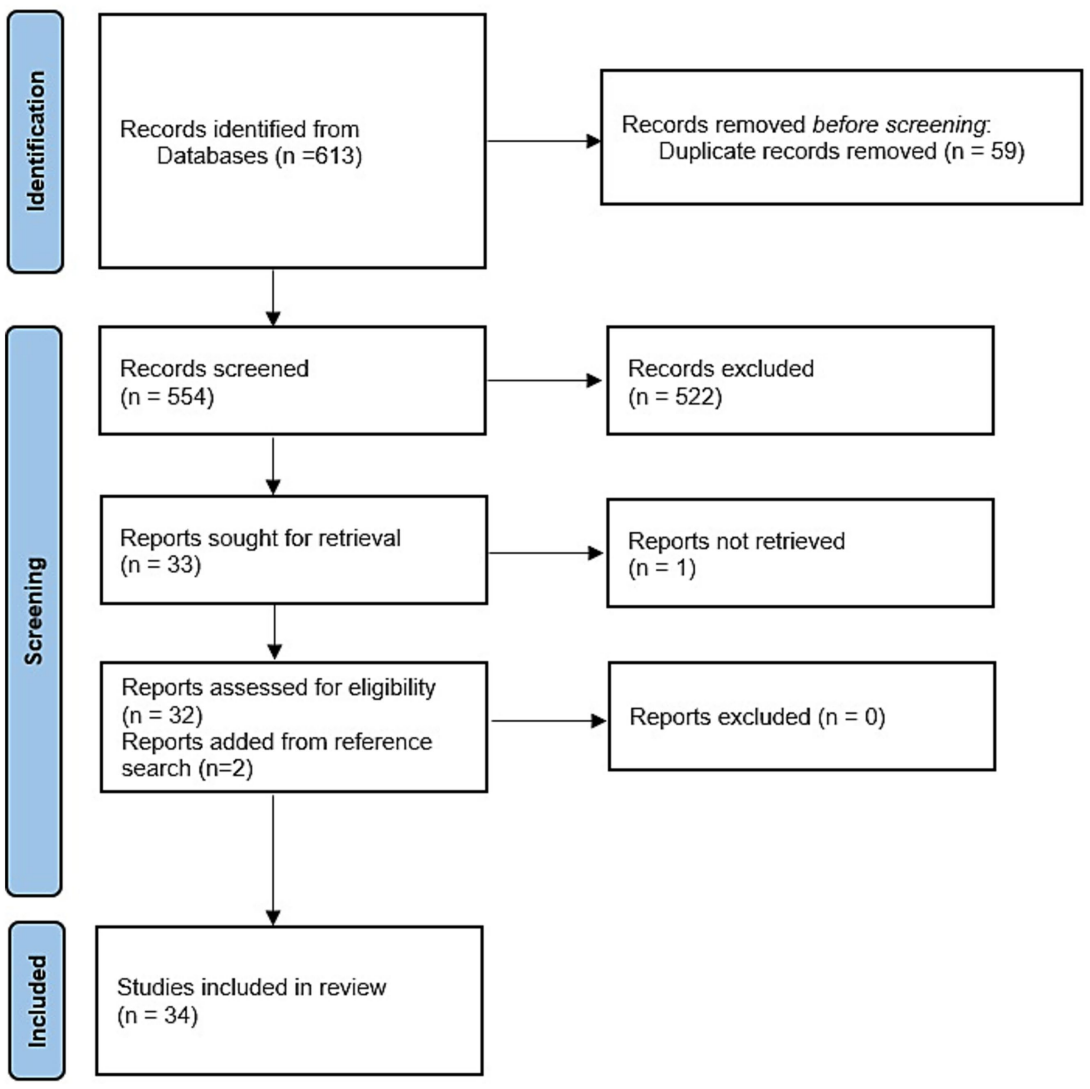
PRISMA flow diagram for studies included in the review.

A total of 34 studies met the inclusion criteria and were thus considered for full paper review and data extraction (Figure 1). Review papers, qualitative papers, and mathematical modeling papers, as well as those outside the scope of the topic, accounted for the majority of the exclusions. A total of 62/554 (11.2%) papers were in a non-English language, with only 1/62 (1.6%) meeting the inclusion criteria. This paper (27), in French, described the seroprevalence of RVF in cattle, sheep, and goats in Niger from samples collected in 2017.

### 3.2 Study characteristics

The peak number of publications occurred in 2022 (7/34; 20.6%), followed by 2018, 2020, and 2021, each contributing 4/34 papers (11.8%). In 2014, 2017, and 2023, 3 /34 papers (8.8%) were published in each year. In 2005, 2006, 2011, 2012, 2016, and 2019, one paper per year (2.9%) was published. The mean number of years between study conduct and publication was 3.4 years (95% CI: 2.9–3.9).

**Figure 2:**
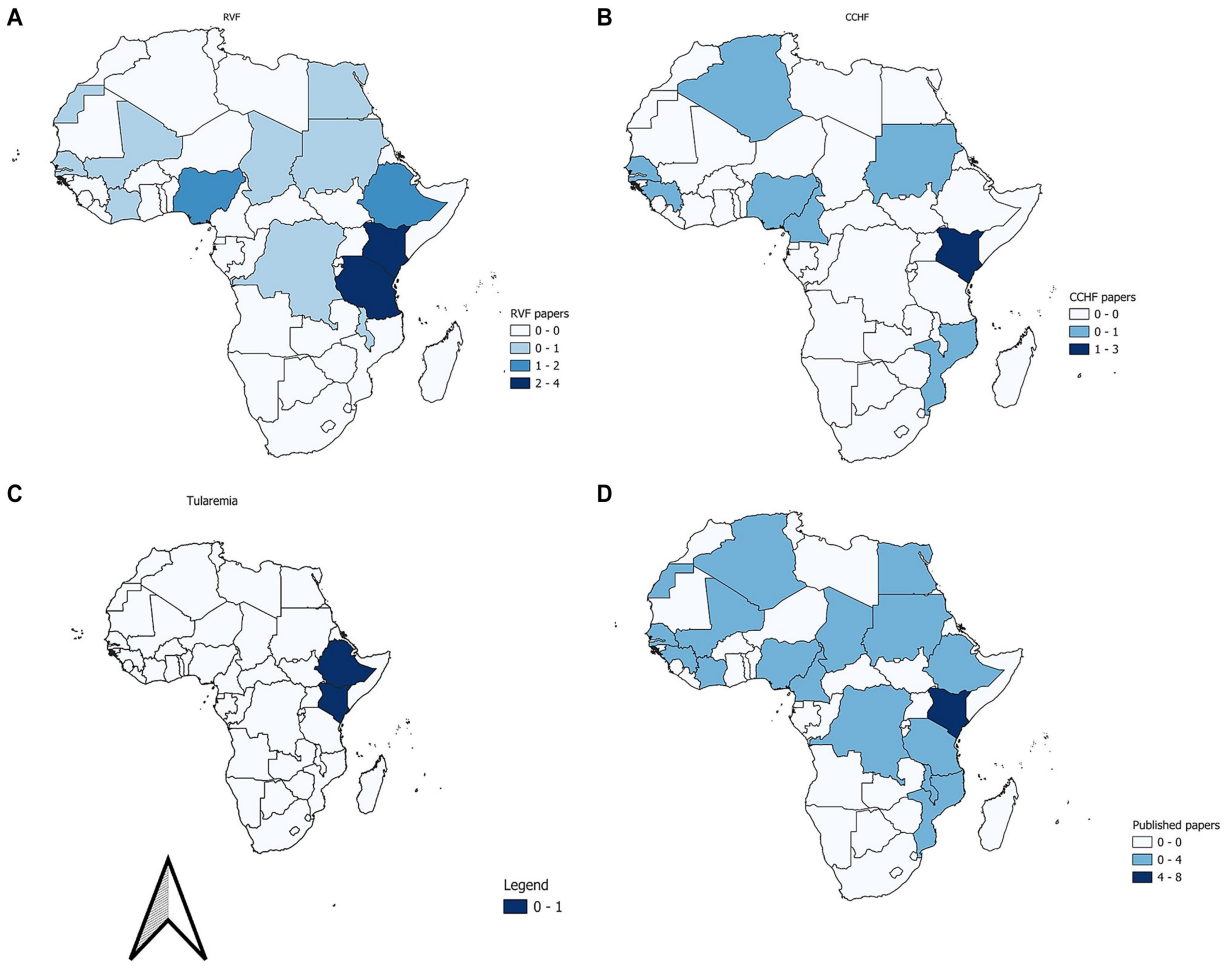
Distribution of RVF **(A)**, papers on CCHF **(B)**, *Ft* **(C)**, and all papers included **(D)** in Africa for the period 2004 to 2024.

A total of 17/52 (32.7%) countries in Africa were included in this study. Kenya had the highest number of published papers at 8/34 (23.5%), followed by Tanzania (4/34; 11.8%), Nigeria (3/34; 8.8%), Senegal (3/34; 8.8%), Ethiopia (3/34; 8.8%), and Sudan (2/34; 5.9%). Algeria, Cameroon, Chad, Democratic Republic of Congo, Egypt, Guinea, Ivory Coast, Malawi, Mali, Western Sahara, and Mozambique have 1/34 (2.9%) papers each (Figure 2).

A total of 22/34 (64.7%) papers investigated the RVF, while 10/34 (29.4%) papers investigated CCHF. Two (5.9%) papers investigated tularemia.

In all papers, the animal production node was investigated by 27/34 (79.4%) studies, with the consumer node of value chains being investigated by 11/34 (32.4%) of the papers. The animal product trader/butcher/middleman/herdsman node was investigated by 4/34 (11.8%) papers. A total of 23/34 (67.6%), 6/34 (17.6%), and 5/34 (14.7%) of the papers reported prevalence, incidence, and positivity as their effect measure, respectively.

### 3.3 Risk of bias assessment

**Figure 3:**
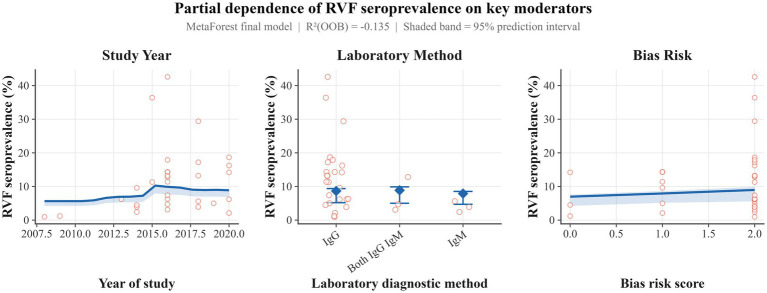
Plots showing the effect of the study year **(A)**, lab method **(B)**, and bias risk **(C)** on the RVF prevalence. Only the study year was found to be significant.

On bias assessment, all studies included underwent peer review and used a laboratory method to test samples (Figure 3). A total of 28/34 (87.5%) of the papers utilized enzyme-linked immunosorbent assay (ELISA) methodology, while 4/34 (9.4%), 1/34 (2.9%), and 1/34 (2.9%) used polymerase chain reaction (PCR), western blot, and serum neutralization test (SNT), respectively. For papers that used ELISA, 25/28 (89.3%) tested for immunoglobulin G (IgG), while 2/28 (7.1%) tested for Immunoglobulin M (IgM). Only one (7.1%) paper tested for both. A total of 14/34 (41.2%) of the papers had sample size calculation and random selection of participants, while 8/34 (23.5%) had either of the two. A total of 12/34 (35.3%) of the papers had neither. A summary of all papers included is shown in Supplementary Table 1.

### 3.4 Combined occurrence estimates

The overall corrected prevalence for RVF amongst people associated with livestock value chains from five reported studies was 29% (95% CI: 7–69%, prediction interval: 0–99%). Before correction, there was potential publication bias (Egger’s test; t = −4.46, DF = 3, *p* = 0.02). This corrected model showed good heterogeneity [I^2^ = 99.1% (95% CI: 98.8–99.3%)]. Stratifying this according to people’s roles in the value chain, a total of five papers investigated RVF prevalence on the consumer node of value chains. The estimated prevalence was 12% (95% CI: 4–31%, prediction interval: 0–90%). Heterogeneity was estimated at 98.8% (95% CI: 98.2–99.1), and this was significant (Q = 320.25, DF = 4, *p* < 0.0001). Furthermore, two studies were available for the butcher/meat trader/middleman/ herdsman node investigating the RVF prevalence. The pooled prevalence from this node was 10% (95% CI: 0–74%, prediction interval: 0–100%). Heterogeneity was I^2^ = 99.3% (95% CI: 98.7–99.6) and was significant (Q = 140.38, DF = 1, *p* < 0.0001).

All animals tested for were placed in the production node of the livestock value chain. The corrected (Egger’s test; t = −4.52, DF = 33, *p* < 0.0001) pooled prevalence was 13% (95% CI: 10–16%, prediction interval: 1–64%). This corrected model had an I^2^ of 94.4% (95% CI: 93.3–95.3), and this heterogeneity was significant (Q = 836.38, DF = 47, *p* < 0.0001).

For cattle, the corrected pooled prevalence was 16% (95% CI: 10–26%; prediction interval: 1–72%). The pooled prevalence was corrected due to a significant Egger’s test result (t = −3.2, DF = 9, *p* = 0.01). This model had an I^2^ = 92.8% (95% CI: 89.9–94.8%). Two studies also reported incidence of RVF in cattle with the overall incidence being 0.07 (95% CI: 0.02–0.26; prediction interval: 0.0–1.0) per animal year with a heterogeneity I^2^ = 99.7% (95% CI: 99.6–99.8%; *p* < 0.0001).

For sheep, there was significant publication bias (t = −3.02, DF = 4, *p* = 0.0393), and after correction, the overall prevalence was 13% (95% CI: 5–30%; prediction interval: 0 to 88%) with an I^2^ of 93.3% (95% CI: 89.1–95.9%) which was significant (*p* < 0.0001). Furthermore, four studies reported incidence of RVFV in sheep with the overall incidence being 0.06 (95% CI: 0.04–0.09): prediction interval 0.01–0.24 per animal year with a heterogeneity I^2^ = 96.0% (95% CI: 92.6–97.9%; *p* < 0.0001).

In goats, the summary prevalence was 4% (95% CI: 2–9%; prediction interval: 0–37%), based on six studies. Heterogeneity in the model was I^2^ = 91.1% (95% CI: 83.5–95.2%, *p* < 0.001). Moreover, four studies reported incidence of RVFV in goats with the overall incidence being 0.06 (95% CI: 0.04–0.09; prediction interval: 0.01–0.29) per animal year with a heterogeneity I^2^ = 97.9% (95% CI: 96.4–98.7%; *p* < 0.0001). Among camels, the pooled prevalence from four papers was 10% (2–38%, prediction interval: 0–98%). Estimated heterogeneity was 98.1% (95% CI: 96.8–98.8%, *p* < 0.0001).

A total of three studies investigated CCHF in humans, with the pooled prevalence being 6% (95% CI: 2–19%; prediction interval: 0–94%). The model had an I^2^ of 98.7% (95% CI: 97.7–99.2%), which was significant (*p* < 0.0001). A total of three papers investigated CCHFV prevalence on the consumer node of value chains. The pooled prevalence estimated was 9% (95% CI: 3–23%, prediction interval: 0–94%) with significant heterogeneity (*p* < 0.0001). Only one study was available and investigated the prevalence of CCHF in cow owners, slaughterhouse workers and cattle herders with the reported prevalence estimates being 1, 0.5 and 2.3%, respectively (Table 2).

**Table 2:** Pooled effect measures of occurrence of RVF and CCHF compared across different factors.

Factor	Levels	Rift Valley fever virus		Crimean-Congo hemorrhagic fever virus		
		Prevalence (95% CI)	Incidence per animal year (95% CI)	Prevalence (95% CI)	Incidence per animal year (95% CI)	Positivity (95% CI)
Species	Human beings	29% (7–69%)^a^		6% (2–19%)		
	Cattle	16% (10–26%)^a^	0.07 (0.02–0.26)	19% (3%–66)		
	Goats	6% (4–10%)				
	Sheep	13% (6–25%)	0.06 (0.04–0.09)		0.35 (0.31–0.40)	
	Camels	19% (7–43%)		48% (8–91%)		
	Ticks (pools)					2% (0–5%)
Value chain node	Consumer	12% (4–31%)		9% (3–23%)		
	Production	13% (10–16%)^a^		15% (4–44%)		
	Butcher/herdsman/middleman	10% (0–74%)				

a Corrected pooled effect using the trimfill command.

In animals, a total of nine papers investigated the prevalence of CCHF at the production node. A random-effects model yielded a pooled prevalence of 15% (95% CI: 4–44%; prediction interval: 0–98%) with significant heterogeneity (*p* < 0.001).

A total of four studies reported CCHF prevalence in cattle, with the overall prevalence of 19% (95% CI: 3–66%; prediction interval: 0–100%) and significant heterogeneity (*p* < 0.0001).

Whereas only one study investigated the incidence of CCHF in sheep. In this study, the reported incidence was 0.35 (95% CI: 0.31–0.40) per sheep-year. No study reported either the prevalence or the incidence of CCHF in goats. However, for camels, two studies investigated the prevalence of CCHFV, with a pooled prevalence of 48% (95% CI: 8–91%, prediction interval: 0–100%). A Q test of heterogeneity was significant (*p* < 0.0001).

Three studies investigated the positivity of tick pools for CCHF. The pooled effect size was 2% (95% CI: 0 – 5%). There was significant heterogeneity in the studies (*p* < 0.001).

Only two papers investigated tularemia, both of which tested individuals who reported to the hospital with fever. Pooled positivity was 2% (95% CI: 0–8%). This model had significant heterogeneity (Q test *p* < 0.001).

### 3.5 Risk factor analysis

Two papers reported age as a risk factor for RVF positivity in humans. One paper categorized age as below 29 and above 30 years, while the other treated age as a continuous variable. The pooled odds ratio was 2.8 (95% CI: 2.7–2.8; *p* < 0.001). However, the model lacked heterogeneity (Q test *p* = 0.7662).

For gender in humans, three studies reported the odds ratio for RVF. All three studies used females as the reference category. The combined OR from these studies was 5.20 (95% CI: 3.09–8.76), which was significant (*p* < 0.0001). However, this model lacked heterogeneity with an I^2^ = 0.0% (95% CI: 0.0–89.6%; *p* = 0.9381).

Two papers investigated age as a risk factor for RVFV infection in small ruminants. Of these two papers, one categorized age as young/old, while the other treated age as a continuous variable. The pooled odds ratio was significant in the common effects model [OR = 2.99 (95% CI: 2.49–3.59; *p* < 0.0001)] but non-significant in the random effects model [OR = 2.1 (95% CI: 0.7–6.5; *p* = 0.1971)]. This model had significant heterogeneity (Q test *p* < 0.001).

A total of nine studies investigated whether animal sex was a risk factor for RVF. Of these, 5/9 (55.6%) studies in three papers used female sex as the reference category, while 4/9 studies in two papers used male sex as the reference category. For papers with females as the reference base, cattle, sheep, and camels accounted for 1/5 (20%) each, while goats accounted for 2/5 (40%). Camels, sheep, goats, and cattle had ¼ (25%) of their papers with males as the reference category.

Of the two papers that investigated sex of the animal as a risk factor and had females as their base category, the pooled odds ratio was 1.6 (95% CI: 0.7 - 3.8; *p* = 0.3079). The model lacked heterogeneity (Q test *p* = 0.7406). Of the eight papers investigating age as a risk factor, a total of 2/8 (25%) of the papers described the risk factor in camels. Both these papers had differing categorization of age, with one having small/old and the other <5 />5 years. Thus, the calculated pooled odds ratio of 4031.2 (95% CI: 55.0–295508.3; *p* < 0.001) with non-significant heterogeneity (*p* < 0.001) was difficult to interpret.

Two studies reported that mixing animal species was a risk factor for RVF. One study reported mixing sheep and goats, while the other reported having cattle, sheep, and goats in the same herd. The combined odds ratio was 33.34 (95% CI: 0.72–1548.64) with a *p*-value of 0.0734. The model lacked heterogeneity (I^2^ = 0.0% and a tau^2^ of 0). Some of the risk factors were reported in only a single paper and are summarized in Table 3.

**Table 3:** Other risk factors reported by single studies.

Rift Valley fever virus			Crimean-Congo hemorrhagic fever virus		
Risk factor	Levels	OR (95% CI)	Risk factor	Levels	OR (95% CI)
Seasonality affected the development of RVF in camels	Season dry versus wet*	5.36 (1.46 - 19.66)	Tick control	Whether a farmer implements tick control* or not	2.2 (1.1–4.4)
Being a pastoralist	Pastoralists versus others*	2.9 (1.21–6.89)	Breed of camel	Western* or Bushari	6.6 (2.4–18.4)
Distance to water body	Below 8 KM versus above 8 KM*	0.1 (0.02–0.7)	Contact with animals	Individuals who split a carcass versus those who did not*	1.92 (1.05–3.72)
History of abortion in sheep is owned	History of abortion versus no history*	4.3 (1–18.9)	Seasonality	Dry vs. wet* seasons	0.33 (0.15–0.68) in sheep and 0.59 (0.27–1.18) in humans
Contact with animals	Individuals splitting carcass versus those who did not*	10.84 (1.97–71.16)			
Animals are getting sick due to diseases in humans	An increase of 1% in the prevalence of RVF in humans	12.9 (2.8–58.7)			
Contact with animals during birthing	Contact versus no contact*	1.69 (1.14–2.51)			
Contact with cattle	No* versus yes	1.38 (1.01 – 1.89)			
Contact with donkeys	No* versus yes	1.38 (1.14–1.67)			
Use of a mosquito net	Use of a mosquito net versus not using*	0.21 (0.3– 0.88)			
Livestock having RVF	An increase of 1% in the prevalence of RVF in animals	4.28 (1.4–13.6)			

*Reference category.

A total of two out of seven papers reported age as a risk factor (28.6%) among humans for CCHF. Both papers treated age as a quantitative continuous variable. The pooled odds ratio was 2.8 (95% CI: 2.7–2.8; *p* < 0.001). However, this model lacked significant heterogeneity (Q test *p* = 0.9). Two studies investigated the effect of the gender of human participants on their susceptibility to CCHF. The reference categories for both papers were different (male and female, respectively). The pooled odds ratio was 2.01 (95% CI: 1.22–3.32), which was significant (*p* < 0.001). However, the model lacked heterogeneity (Q = 0.86, DF = 1, *p* = 0.3538).

For CCHF, a total of 3/7 (42.9%) papers reported age as a risk factor (42.9%) for cattle. Two of the three papers treated age as a categorical variable with differing categories, while one treated age as a continuous variable. The pooled odds ratio across these three studies was 4.6 (95% CI: 1.89–11.27; *p* = 0.0008) with significant heterogeneity (Q test *p* < 0.001).

A total of 2/8 (25%) papers reported sex as a risk factor for CCHF in cattle. Both papers used different reference categories (male and female for each). The pooled odds ratio was 4.5 (95% CI: 0.23–86.33; *p* = 0.3196).

Two studies examined whether the presence of ticks on animals was a risk factor for infection with CCHF. The random effects pooled OR estimates from a random effects model were 4.5 (95% CI: 0.36–56.5) with a *p* value of 0.2442, while the fixed effects model was 3.15 (95% CI: 1.67–5.93) with a p value of below 0.05. The model lacked heterogeneity (Q = 1.18, DF = 1, *p* = 0.2773).

### 3.6 Moderator analysis

Random forest analysis identified the laboratory method used, study year, and the bias risk as the most relevant predictors of RVF prevalence (Figure 3). The prevalence increased by 0.13 (95% CI: 0.01–0.25) units per year, and this was significant (*p* = 0.0297). The I^2^ of this model was 95.42% with a tau^2^ of 0.6470.

Two moderators were identified by random forest plots as major moderators of CCHF prevalence. These were the species of animal investigated and the country of publication. Including both the number of countries combined with the number of species in a multivariable model would have led to overfitting of the model, since the number of studies reporting prevalence of CCHF (outcome) was small (10). Thus, univariate mixed-effect models were fitted. The prevalence increased by 2.61 (95% CI: −0.39–5.6) units in camels compared with humans. The *p*-value for this was borderline (*p* = 0.07). All other species had non-significant results. The I^2^ of this model was 98.75% with a tau^2^ of 1.7796.

The prevalence of CCHF increased by 2.41 (95% CI: 0.31–4.51) and 1.52 (95% CI: 0.16–2.89) points in Algeria and Cameroon compared to Kenya. It decreased by 2.19 (95% CI: 0.21–4.18) in Guinea compared to Kenya. The *p*-values for these relationships were significant (0.044, 0.45, and 0.45), respectively. Overall, the model for the study country lacked heterogeneity (I^2^ = 0%, Tau^2^ = 0).

## 4 Discussion

Pastoralism is widely practiced in Africa, with pastoralists living at the interface of humans, livestock, wildlife, and the environment and facing a high risk of zoonotic disease spread (28). An understanding of disease risks within these systems is therefore important, especially given that products from these systems are consumed in large areas of the continent. This paper gathered information on three case study zoonotic pathogens (CCHFV, RVFV, and *Ft*) that can spread through livestock value chains. Most studies were for RVFV followed by CCHFV. *Ft* had very few studies indicating that either the disease was poorly studied or that the true prevalence of it is low. Only around a third of the countries in Africa had studies included, despite pastoralism being practiced in many more countries in Africa (1).

Most of the papers utilized ELISA methodology and thus were representative of prior exposure to the pathogens. The highest pooled prevalence for RVF was in human beings. This may be due to the multiple routes of infection compared to animals. These include vectors and contact with contaminated animal products, and the relative lack of disease control measures in pastoralist systems. Among livestock, camels had the highest prevalence, followed by cattle and then sheep. In a review on RVFV prevalence in Africa, Ebogo-Belobo et al. (29) showed a non-significant difference in prevalence estimates for humans and animals, with the animal prevalence being higher.

There was a high prevalence of both RVF and CCHF in camels. Generally, camels are not high in the meat food chain and, together with their high value (for milk, draught, and sociocultural reasons), are long-lived, possibly leading to higher exposures.

There was a difference in the prevalence estimates among the three value chain nodes examined for RVF, although overlapping confidence intervals showed the differences were non-significant. The producer node had the highest prevalence, followed by the consumer node, with the middleman node having the least. The producer node, being the confluence of multiple susceptible species, would have higher transmission rates than other nodes. However, the mosquito vector being found across all nodes for RVF could explain the non-significant differences across nodes. Furthermore, the number of studies in the production node was higher than in other nodes, suggesting the need for further research at that level. In the absence of more data on the impact of RVF on value chain nodes, value chain mathematical models may help enumerate possible differences in transmission rates across nodes.

Prediction intervals inform the range of estimates that are likely to be obtained in similar studies to the pooled ones (30). However, their usefulness is suspect where the number of studies is less than 10 (30, 31) unless Bayesian hierarchical modeling is used (32). In the present study, only the meta-analyses of prevalence estimates for RVF in all animals and RVF in cattle had more than 10 studies. In these cases, the prevalence estimates in 95% of future similar studies could be as low as 1% and as high as 64% (all animals) and 75% (cattle). The wide variation may reflect differences in environmental conditions that favor or limit vector multiplication, with higher prevalences occurring in seasons favorable for vector multiplication.

The *Aedes*, *Culex*, and *Anopheles* mosquito genera are implicated in the spread of RVFV, with epidemics coinciding with environmental conditions favorable for the vectors (33). Two reports analyzed in this review investigated environmental factors, i.e., distance from a water body and seasonality (dry vs. wet seasons). Both were significant. Many of the papers also examined how the locality of the study affected the prevalence of RVF. This indicates the importance of environmental conditions to RVF prevalence and is supported by literature (34).

CCHF is endemic in Africa, with environmental conditions being the greatest drivers of occurrence in an area (35). A systematic review on the occurrence of CCHF in Arab countries found an overall prevalence of 29% in camels, 22% in cattle, and approximately 10% in ticks, compared to 48, 19, and 2% respectively, in this study (36). The prevalence of CCHF in camels was markedly higher in our study, but comparable to that in cattle and ticks.

Age was a significant risk factor in the meta-analysis for CCHF. This may be due to most of the included studies relying on IgG antibody detection. These antibodies have been shown to persist for at least 8 years in human survivors of CCHF (37) and are used most often as indicators of prior infection. Furthermore, division of labor in pastoral settings, including animal herding, is influenced by age (56), which may contribute to differential risk among age groups. However, differing classifications on age, with some papers treating it as a categorical variable while others as continuous, make it difficult to interpret the pooled estimates.

While gender of human beings was significantly associated with CCHF positivity, the differing reference classes for age made it difficult to interpret results. Of the two papers reporting this, the paper by Mhamadi et al. (38) had a sample size of 364 febrile patients, while the study by Lwande (57) had 517 patients in a pastoral area with febrile illness presenting to a hospital. In both studies, there were more women than men. Thus, the significance of gender may have been due to gendered risks and health-seeking behavior, as has been explored before for zoonotic diseases (39–41).

The non-significance of ticks as a risk factor in the random-effects model may have been due to few studies reporting it, and the two studies that did report it being similar (low heterogeneity). Due to the low heterogeneity the fixed-effects model was chosen as the best representation with it reporting that animals with ticks were 3 times more likely to be positive for CCHFV than those without. One of the two studies (42) investigated camels in Sudan and relied on animal owners identifying whether ticks were present on animals or not, and then assessing whether this was a risk factor. The other paper (38) investigated tick infestation in sheep in Senegal. In this study, tick infestation was confirmed by the study personnel, and ticks were also tested for CCHFV after collection.

Pooled prevalence of CCHF was higher in production animals than humans at the consumption node but not significantly. Human beings may be infected either through bites of infected ticks or through direct contact with animal products from infected animals. Ticks attach for a greater time in animals than humans, with attachment time and amount of saliva introduced being major determinants of infection (43). This, together with practices at animal product preparation such as cooking, which heat denatures the virus before eating, may explain the lower prevalence in humans. A lower prevalence in humans than in animals for past infections was also found in another systematic review (44).

Only two papers on *Ft* were included. Tularemia is considered rare in Africa. However, a case report in the southern part of South Sudan, which is a pastoralist area, shows that the pathogen may be present in these populations (45). Furthermore, the bacteria have been detected in wild hares in Algeria (46) and bedbugs in Madagascar (47). A study in Egypt found evidence of Francisella-like endosymbionts in the camel tick *Hyalomma dromedarii* and Ft antibodies in camel abattoir workers (48). Moreover, Ft was confirmed in a French tourist who had travelled to Egypt and participated in outdoor activities with camels (49). The current study finds that about 2% of generalized fevers in pastoralist areas in Africa may be due to *Ft*. Evidence of Francisella has also been found in *Hyalomma dromedarii* in the United Arab Emirates and Saudi Arabia (50, 51), which is especially important given that imports of camels to these countries from pastoralist areas in East Africa occur regularly (52, 53). There is a need for further epidemiological studies on the disease, including in animals and vectors, to better understand its occurrence in Africa.

Disease control efforts for RVF in Africa mainly consist of vaccination with live attenuated or inactivated vaccines (in animals), community education, vector control, quarantines, and animal product control (e.g., banning of slaughter). However, underinvestment in these control measures especially in pastoralist production systems hampers their effectiveness and outbreaks are common during favorable environmental conditions. CCHF control focuses primarily on community education, quarantines, animal product control, and tick control. Vaccines for both CCHF and tularemia are under development, but none have been licensed (54, 55).

## 5 Limitations

Our study was not able to specifically characterize value chain nodes, but rather placed RVF and CCHF papers in their most appropriate node. However, this may show that value chain nodes may be important in the spread of diseases, and more research on their explicit role is recommended. The meta-analysis for RVF left out one non-English published paper. Finally, the differences in categorization of continuous variables as well as the choice of reference categories during risk factor analysis made pooling of the results difficult.

## 6 Conclusion

This review investigated the occurrence and risk factors of RVF, CCHF, and tularemia in pastoral setups as reported in published research in Africa for the previous 20 years and their occurrence within livestock value chains. It adequately represents the picture of CCHF, RVF, and tularemia in pastoralist systems in Africa by comprehensively analyzing published records of these diseases. Our results showed that RVFV, CCHFV, and *Ft* are actively circulating in pastoralist systems, with the animal production node having the highest occurrence. While *Ft* may currently be considered rare/absent in Africa, according to the limited available literature, some of the undifferentiated fevers reported in pastoral setups may be due to the disease. This review showed that zoonotic diseases including those previously considered rare and with potential for downstream spread are present in pastoralist production systems. While it may be difficult to provide animal health investments to the pastoralist production system due to constant movement and wide geographical areas, there is a need to develop and fund disease control measures designed for the production systems.

## Data Availability

The raw data supporting the conclusions of this article will be made available by the authors, without undue reservation.

## Footnotes

1
https://www.rayyan.ai/
