# Supplementary material for: Systematic review and meta-analysis for the occurrence of Crimean-Congo hemorrhagic fever, Tularemia, and Rift Valley fever in pastoralist systems in Africa
Source: Front Vet Sci. 2026 Mar 31;12:1624748. doi: 10.3389/fvets.2025.1624748 (PMC13076134; doi:10.3389/fvets.2025.1624748)
Supplement: Supplementary file 1 [file Table_1.DOCX]

Supplementary Material

1. **Supplementary tables and figures**
   1. **Supplementary Tables**

**Supplementary table 1:** Summary of papers included in the study

| Paper | Sample size | Species investigated | Disease | Country | Effect measure | Lab method | SS calculation and randomization | Value chain Node |
| --- | --- | --- | --- | --- | --- | --- | --- | --- |
| (Abdallah *et al.*, 2016) | 240 | Camels | RVF | Sudan | prevalence | ELISA | Random sampling only | Production |
| (Ahmed *et al.*, 2018) | 751 | Humans | RVF | Tanzania | prevalence | ELISA | None | Consumer |
| (Alhaji *et al.*, 2018) | 97 | Cattle | RVF | Nigeria | prevalence | ELISA | Both | Production |
| (Alhaji *et al.*, 2020) | 97 | Cattle | RVF | Nigeria | prevalence | ELISA | Both | Production |
| (Chevalier *et al.*, 2005) | 610 | Sheep and goats | RVF | Senegal | Incidence (t=5 months) | SNT | Sample size calculation only | Production |
| (Di Nardo *et al.*, 2014) | 982 | Camels/ sheep/goats | RVF | Western Sahara | prevalence | ELISA | Both | Production |
| (Dione *et al.*, 2022) | 304 | Cattle | RVF | Mali | prevalence | ELISA | Both | Production |
| (Durand *et al.*, 2020) | 70* | Goats | RVF | Senegal | prevalence | ELISA | Random sampling only | Production |
|  | 429* | Sheep |  |  |  |  |  |  |
|  | 161* | Cattle |  |  |  |  |  |  |
| (Endale *et al.*, 2021) | 397 | Cattle | RVF | Ethiopia | prevalence | ELISA | Sample size calculation only | Production |
| (Georges *et al.*, 2018) | 450 | Cattle | RVF | Democratic Republic of Congo | prevalence | ELISA | Both | Production |
| (Glanville *et al.*, 2021) | 215 | Cattle | RVF | Tanzania | Incidence (t=23 months) | PCR | None | Production |
| (Ibrahim *et al.*, 2021) | 190 | Humans | RVF | Ethiopia | prevalence | ELISA | Both | Consumer and Production |
|  | 108 | Cattle |  |  |  |  |  |  |
|  | 141 | Camels |  |  |  |  |  |  |
|  | 252 | Goats |  |  |  |  |  |  |
|  | 229 | Sheep |  |  |  |  |  |  |
| (Kainga *et al.*, 2022) | 857 | Cattle | RVF | Malawi | prevalence | ELISA | Both | Production |
|  | 518 | Goats |  |  |  |  |  |  |
| (Kanouté *et al.*, 2017) | 192 | Cattle | RVF | Côte d Ivoire | prevalence | ELISA | Both | Production |
|  | 333 | Sheep |  |  |  |  |  |  |
| (Kimbita, Kassuku and ..., 2006) | 186 | Goats | RVF | Tanzania | Incidence (t= 12 months) | ELISA | None | Production |
|  | 38 | Sheep |  |  |  |  |  |  |
| (Mbotha *et al.*, 2018) | 182 | goats | RVF | Kenya | Incidence (t=10 months) | ELISA | Random sampling only | Production |
|  | 65 | Sheep |  |  |  |  |  |  |
| (Muturi *et al.*, 2021) | 120 | Camels | RVF | Kenya | prevalence | ELISA | None | Production |
| (Owange *et al.*, 2014) | 1396 | Cattle | RVF | Kenya | Incidence (t=11 months) | ELISA | Both | Production |
| (Özcelik *et al.*, 2023) | 954 | Humans | RVF | Chad | prevalence | ELISA | Both | Consumer and Production |
|  | 152 | Goats |  |  |  |  |  |  |
|  | 370 | Cattle |  |  |  |  |  |  |
|  | 349 | Sheep |  |  |  |  |  |  |
| (Sindato *et al.*, 2022) | 664 | Humans | RVF | Tanzania | Prevalence | ELISA | Random sampling only | Consumer, production and middleman |
|  | 361 | Cattle |  |  |  |  |  |  |
|  | 394 | Goats |  |  |  |  |  |  |
|  | 242 | Sheep |  |  |  |  |  |  |
| (Tigoi *et al.*, 2020) | 1210 | Humans | RVF | Kenya | prevalence | ELISA | Both | Consumer and middleman node |
| (Horton *et al.*, 2014) | 161 | Cattle | RVF | Egypt | prevalence | ELISA | None | Production |
|  |  |  | CCHF |  |  |  |  |  |
| (Blanco-Penedo *et al.*, 2021) | 148 | Cattle | CCHF | Kenya | prevalence | ELISA | Both | Production |
| (Dzikwi-Emennaa *et al.*, 2022) | 184 | Cattle | CCHF | Nigeria | prevalence | ELISA | Both | Production |
| (Gordon *et al.*, 2022) | 56 | Cattle | CCHF | Cameroon | prevalence | ELISA | Both | Production |
| (Guidoum *et al.*, 2023) | 294 | Camels | CCHF | Algeria | prevalence | ELISA | None | Production |
| (Lwande *et al.*, 2012) | 517 | Humans | CCHF | Kenya | prevalence | ELISA | None | Consumer |
| (Mhamadi *et al.*, 2022) | 364 | Humans | CCHF | Senegal | Positivity | ELISA | None | Consumer and production |
|  | 808 | Ticks |  |  | Prevalence |  |  |  |
|  | 34 | Sheep |  |  | Incidence (t=13 months) |  |  |  |
| (Naidenova *et al.*, 2020) | 882 | Human | CCHF | Guinea | Positivity | PCR | None | Consumer and Production |
|  | 2207 | Ticks |  |  |  |  |  |  |
| (Oludele *et al.*, 2023) | 218 | Humans | CCHF | Mozambique | prevalence | ELISA | Sample size calculation only | Consumer and middleman |
| (Sang *et al.*, 2011) | 1144 pools | Ticks | CCHF | Kenya | Positivity | PCR | None | Middleman |
| (Suliman *et al.*, 2017) | 361 | Camels | CCHF | Sudan | prevalence | ELISA | Random sampling only | Production |
| (Njeru *et al.*, 2017) | 730 | Humans | Tularemia | Kenya | Positivity | Western Blot | None | Consumer |
| (Ramos *et al.*, 2019) | 394 | Humans | Tularemia | Ethiopia | Positivity | PCR | None | Consumer |

1. *Only data on Nomadic pastoralists used

**1.2 Supplementary Figures**


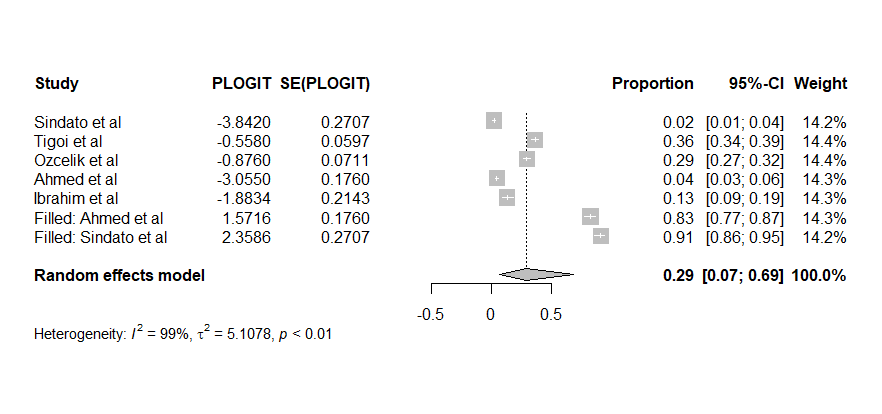


**Supplemental Figure 1:** RVF prevalence in human beings


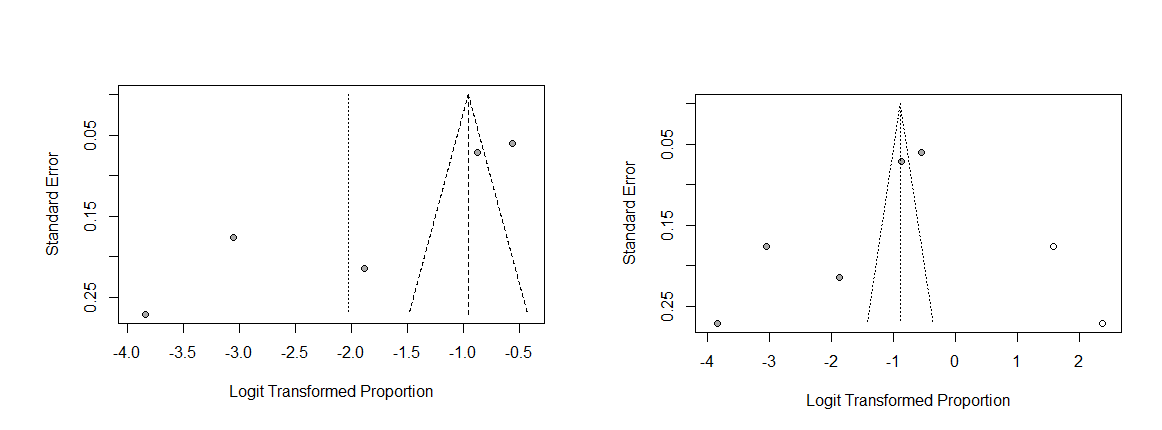


**Supplemental Figure 2:** Uncorrected (left) and corrected funnel plots for RVF prevalence in human beings with filled in studies shown as empty dots


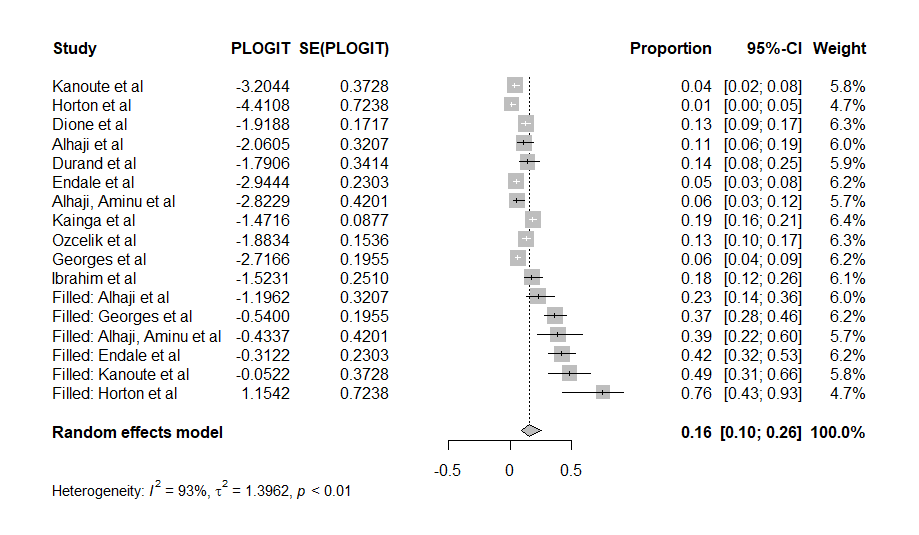


**Supplemental Figure 3:** RVF prevalence in cattle


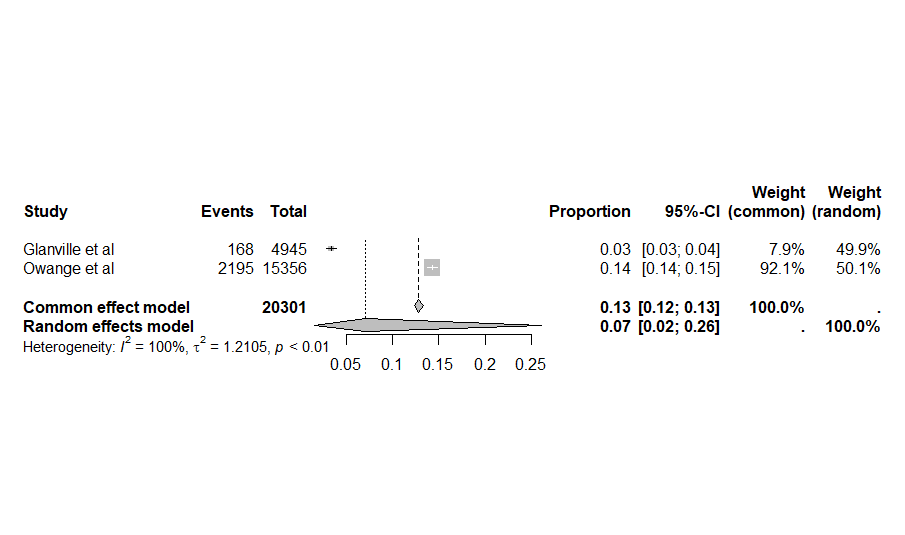


**Supplemental Figure 4:** Forest plot showing the reported incidence of RVF in cattle


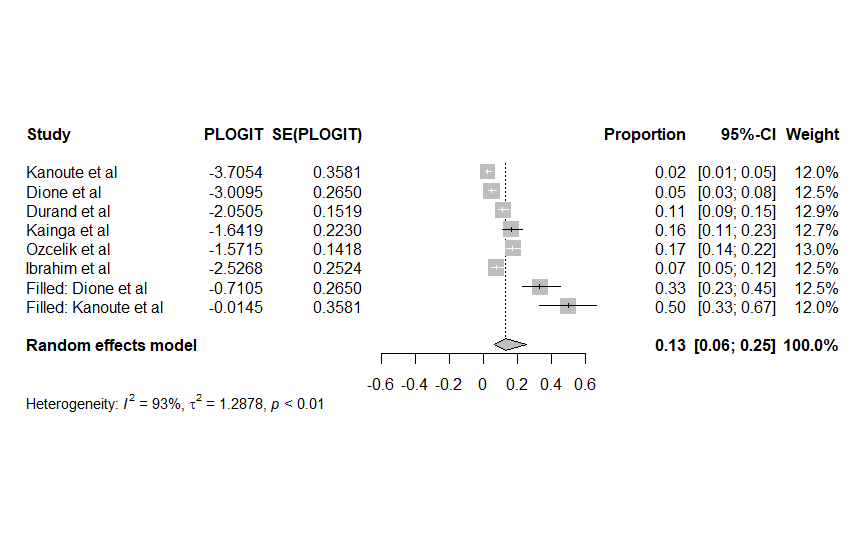


**Supplemental Figure 5:** RVF prevalence in sheep


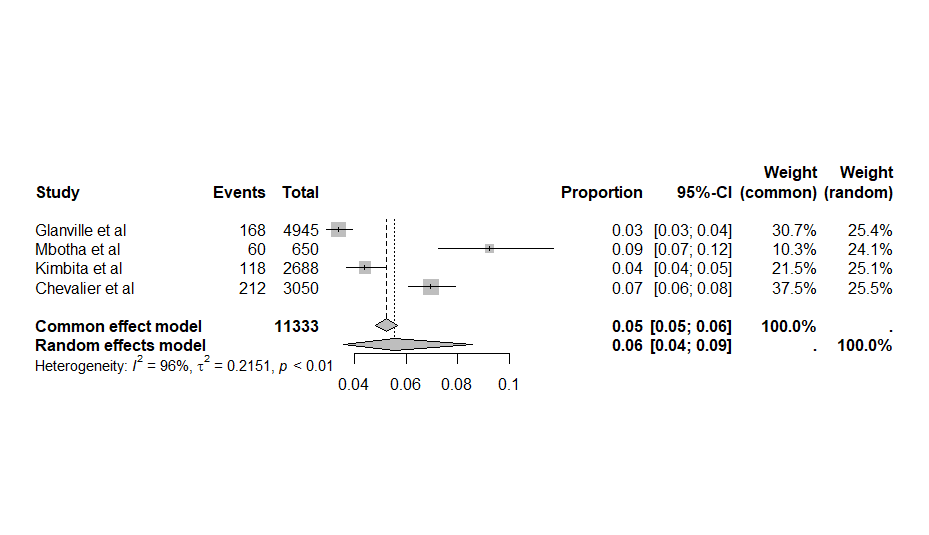


**Supplemental Figure 6:** Forest plot showing the reported incidence of RVF in sheep


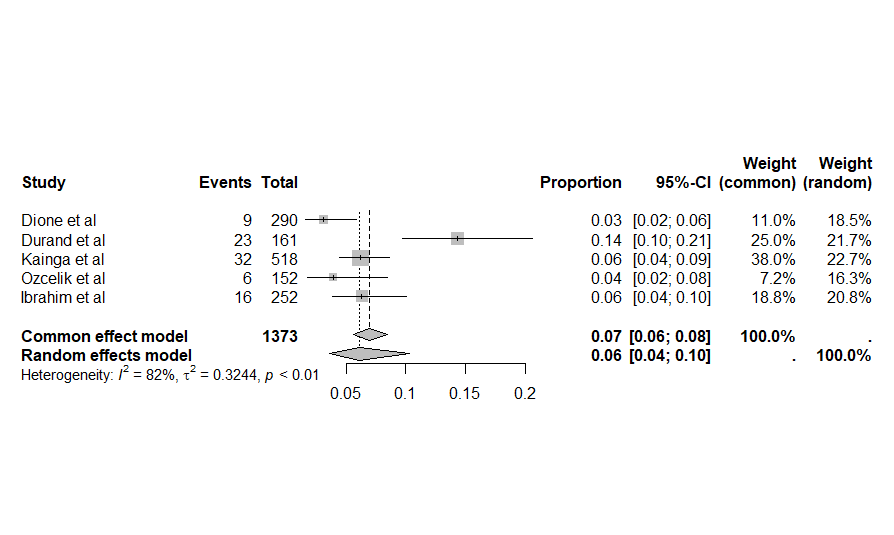


**Supplemental Figure 7:** RVF prevalence in goats


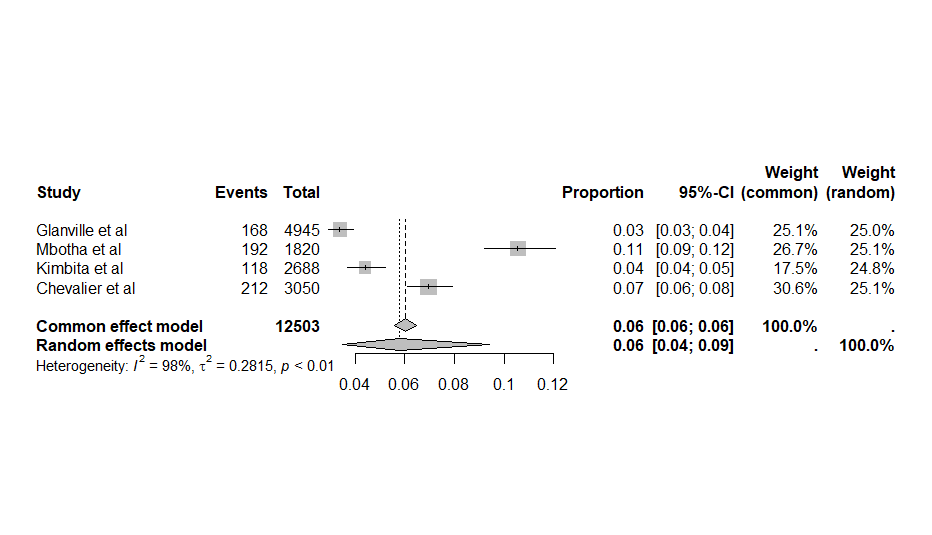


**Supplemental Figure 8:** Forest plot showing the reported incidence of RVF in goats


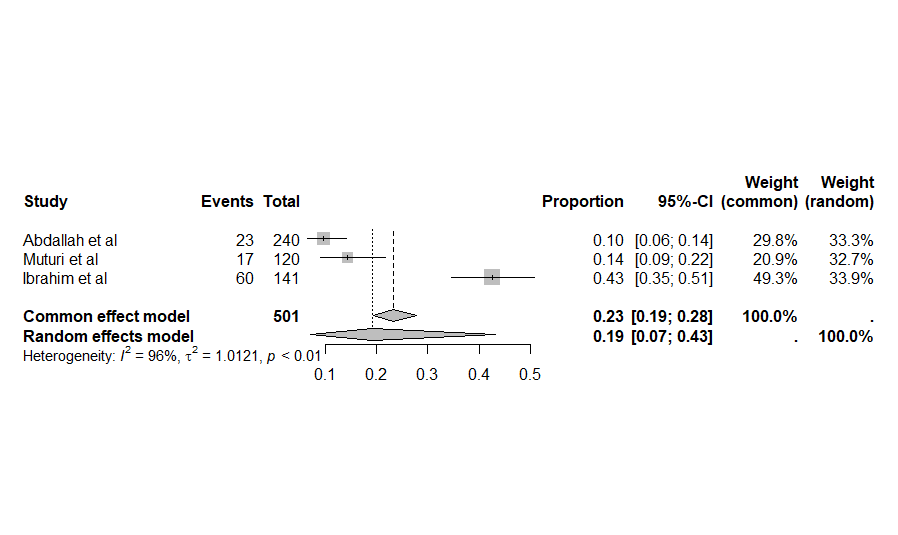


**Supplemental Figure 9:** RVF prevalence in camels


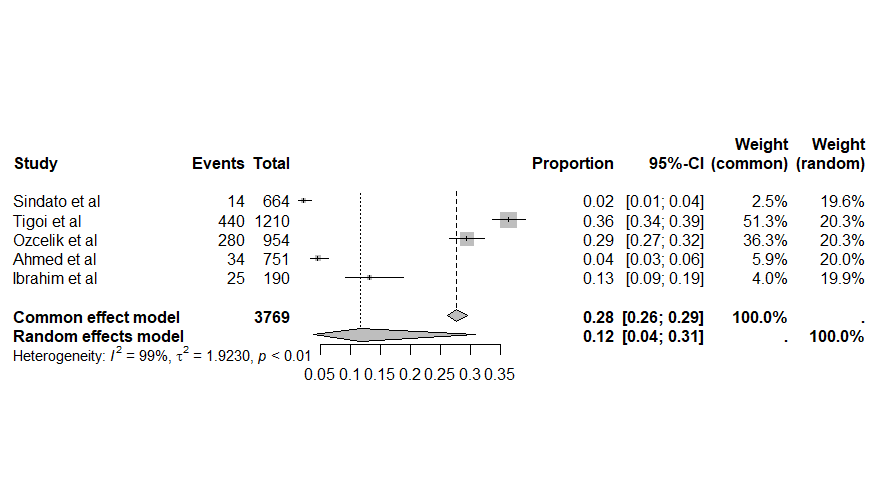


**Supplemental Figure 10:** RVF prevalence reported in the consumer node of value chains


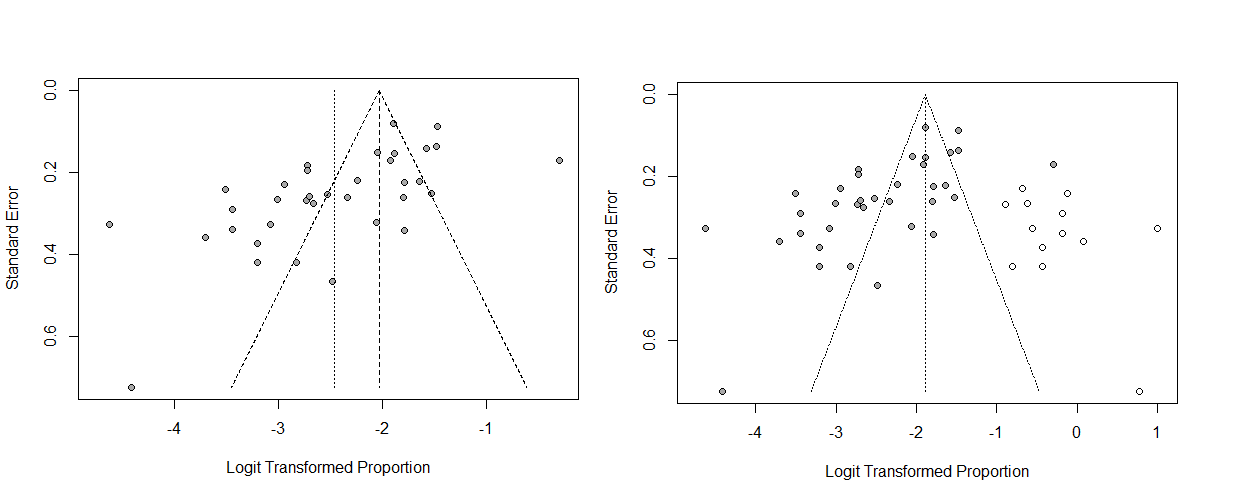


**Supplemental Figure 11:** Funnel plot for prevalence of RVF in the production node of a livestock value chain before (left) and after (right) correcting for publication bias.


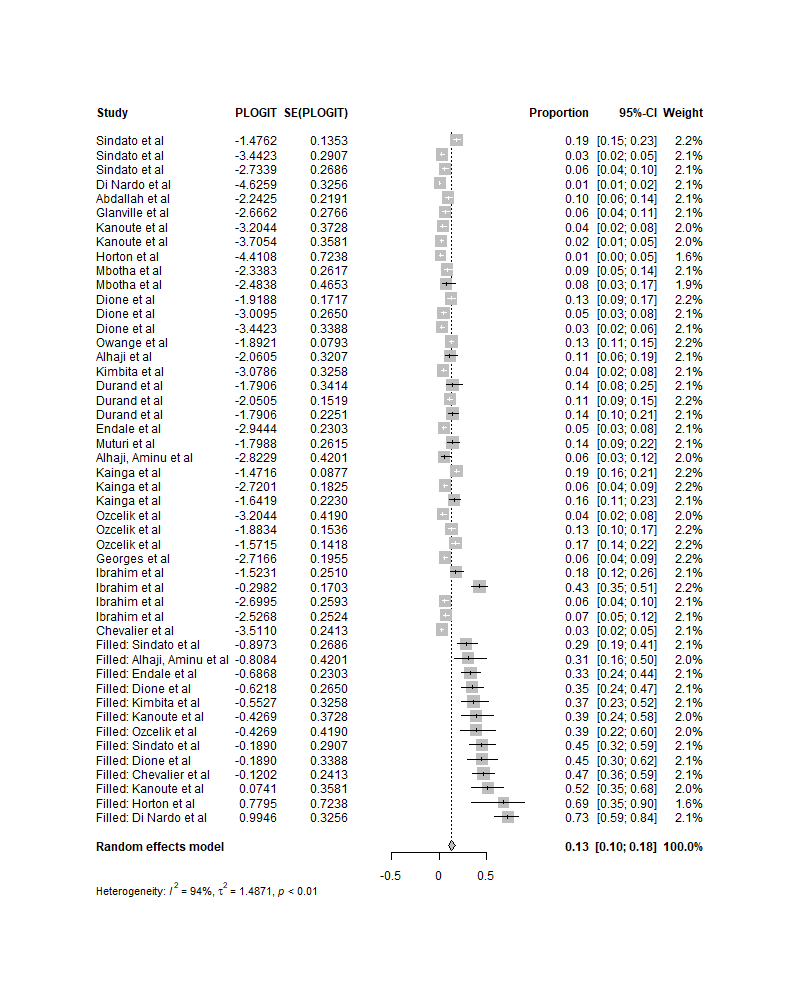


**Supplemental Figure 12:** Reported and corrected prevalence of RVF in the production node of value chains.


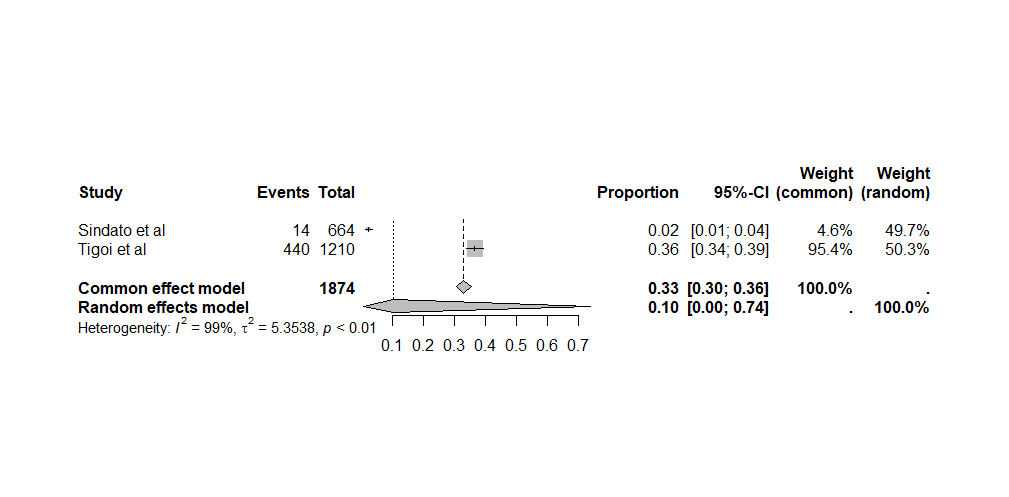


**Supplemental Figure 13:** Reported prevalence of RVF in the butcher/ meat trader/middle man/ herdsman node


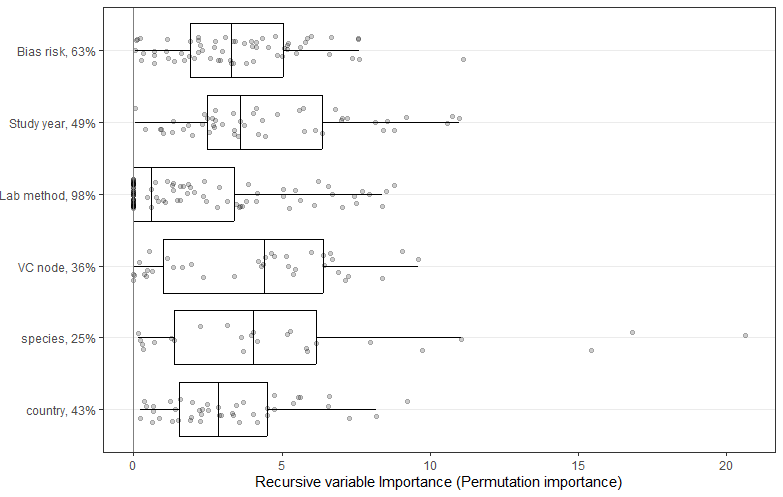


**Supplemental Figure 14:** Box plot showing variable importance recursed 100 times (dots).


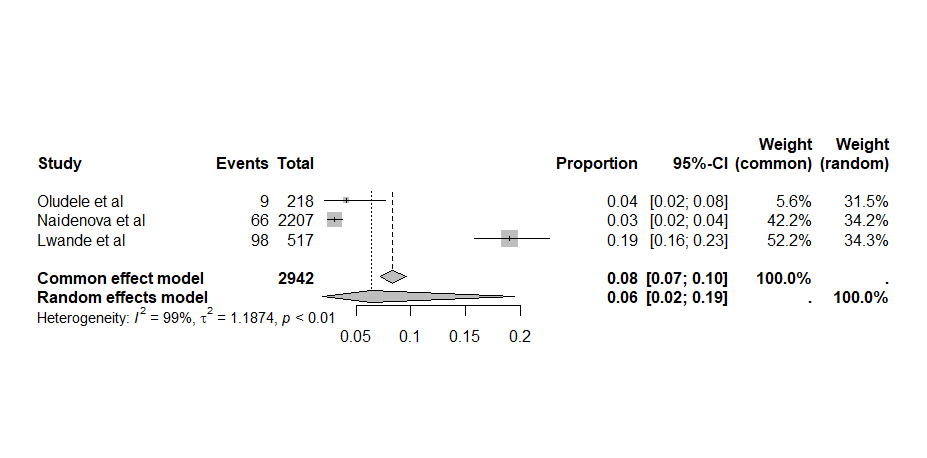


**Supplemental Figure 16:** Prevalence of CCHF in humans


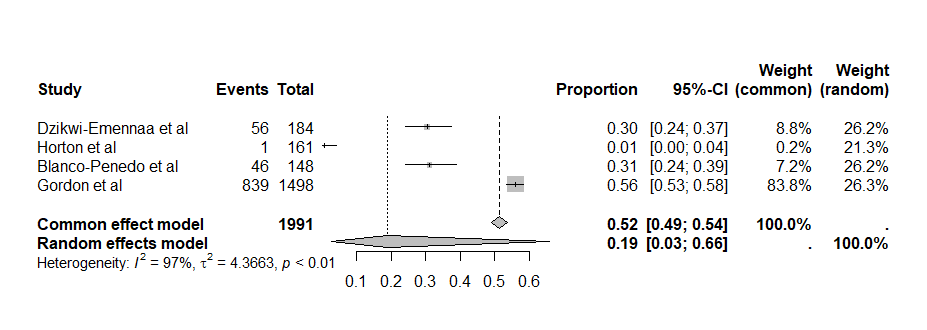


**Supplemental Figure 17:** Prevalence of CCHF in cattle


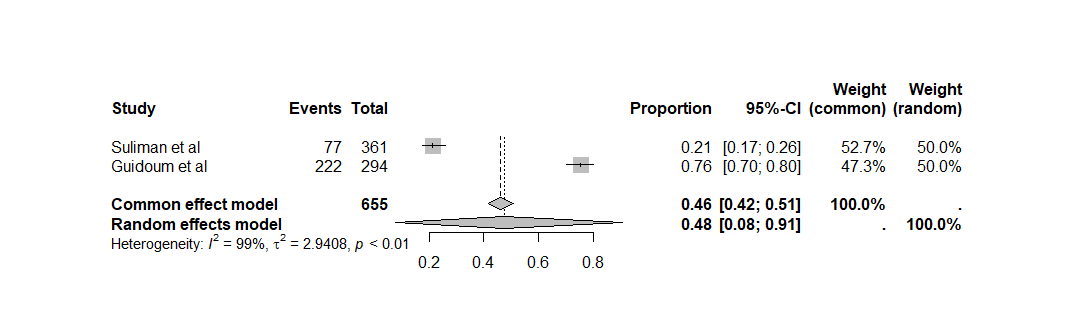


**Supplemental Figure 18:** CCHF prevalence in camels


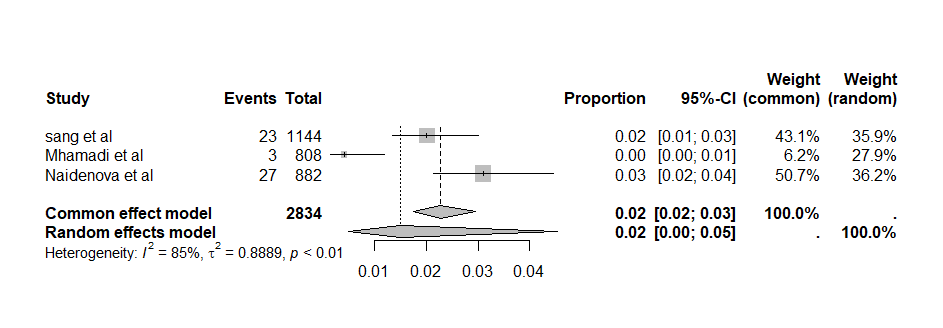


**Supplemental Figure 19:** CCHF prevalence in tick pools


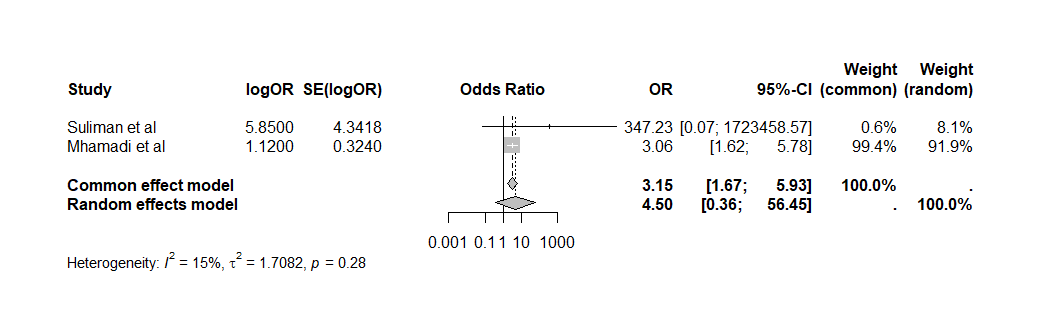


**Supplemental Figure 21:** Presence of ticks on an animal as a risk factor for CCHF


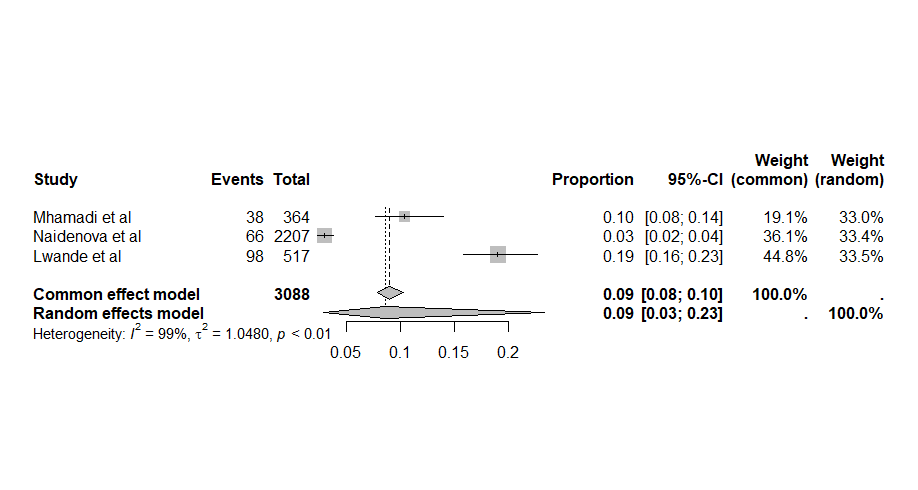


**Supplemental Figure 24:** Prevalence of CCHF in the consumer node of value chains


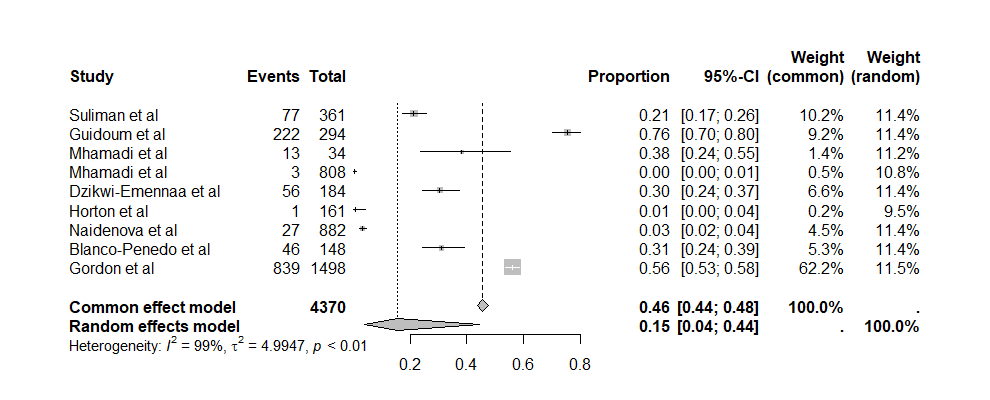


**Supplemental Figure 25:** Prevalence of CCHF in the production node of value chains


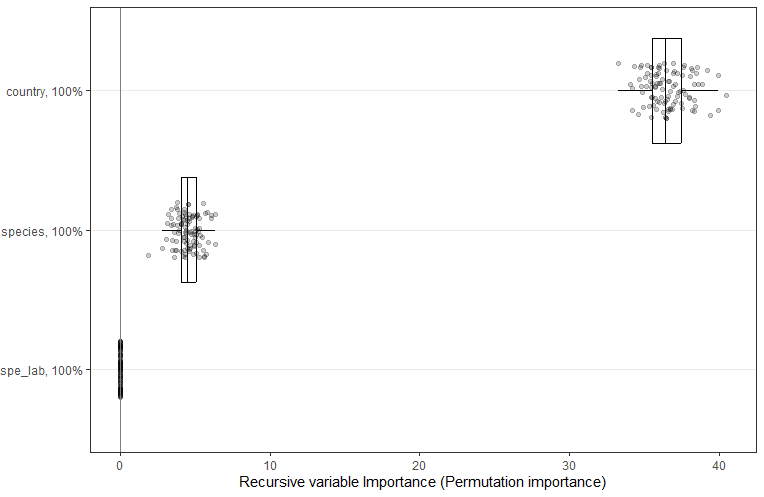


**Supplemental Figure 26:** Box plot showing variables importance recursed 100 times (dots).


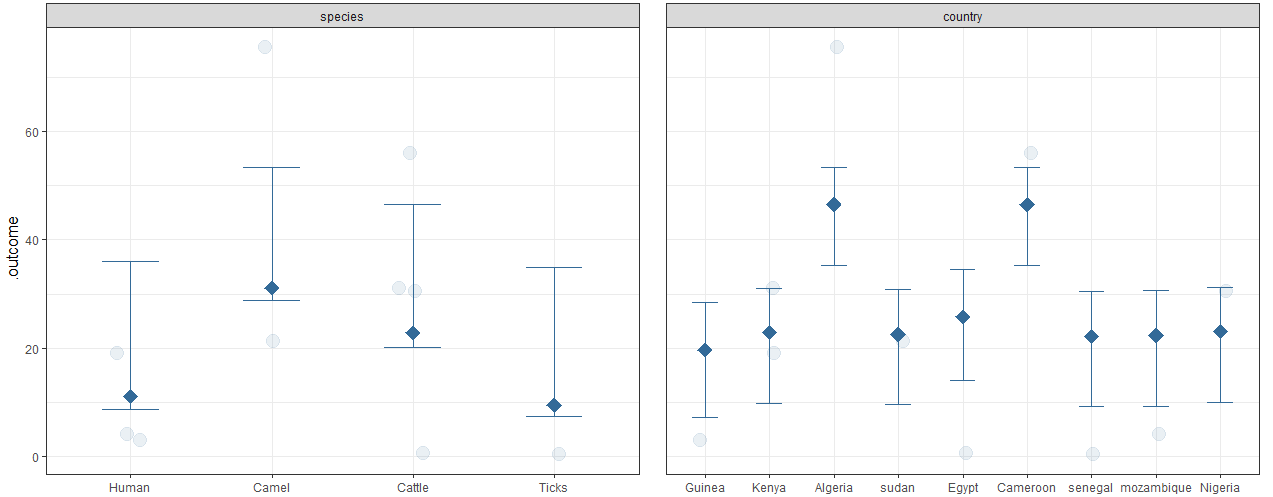


**Supplemental Figure 28:** Final plots showing the effect of species and country risk on the CCHF prevalence using random forest plots.


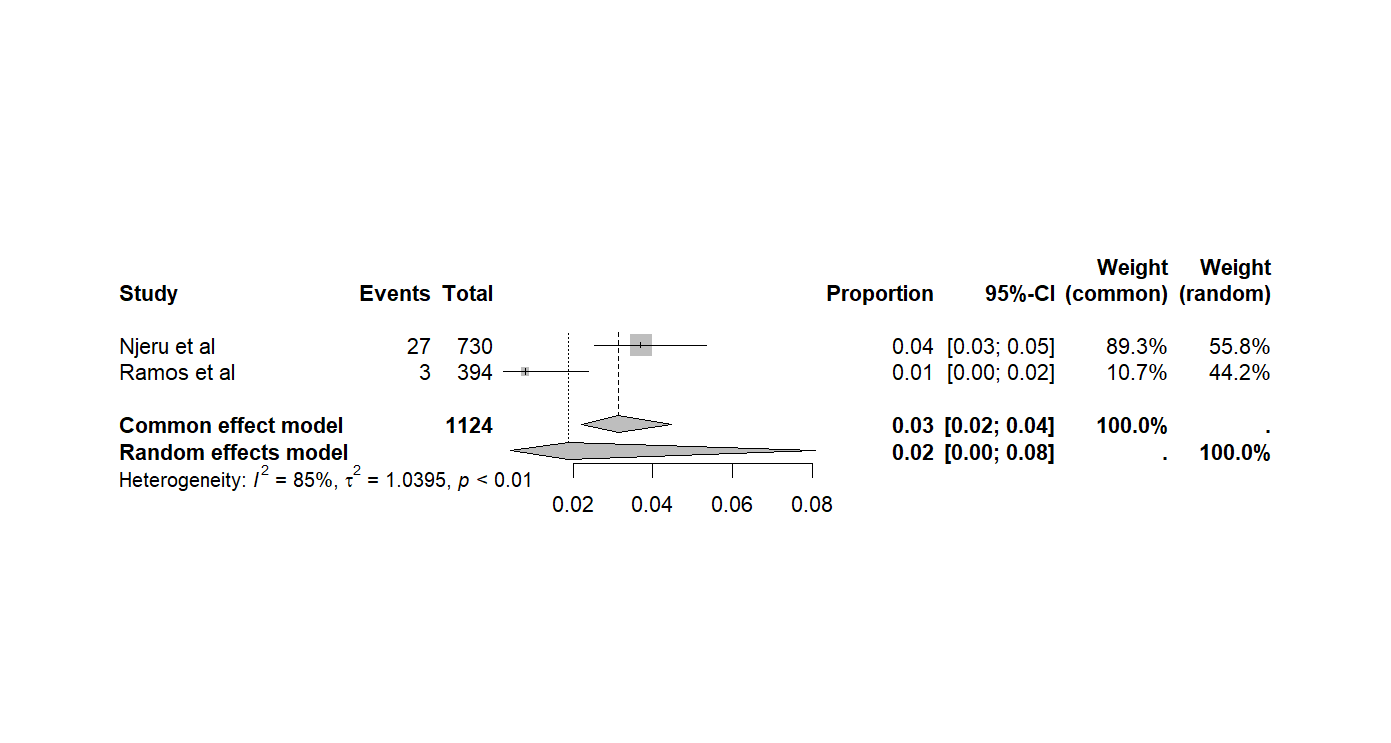


**Supplemental Figure 29:** Forest plot for Tularemia positivity in the two included studies
